# Advances on Innate Immune Evasion by Avian Immunosuppressive Viruses

**DOI:** 10.3389/fimmu.2022.901913

**Published:** 2022-05-12

**Authors:** Hongnuan Wang, Wei Li, Shijun J. Zheng

**Affiliations:** ^1^ Key Laboratory of Animal Epidemiology of the Ministry of Agriculture, College of Veterinary Medicine, China Agricultural University, Beijing, China; ^2^ Department of Preventive Veterinary Medicine, College of Veterinary Medicine, China Agricultural University, Beijing, China

**Keywords:** avian viruses, immune evasion, innate immunity, PRRs, IFNs-I

## Abstract

Innate immunity is not only the first line of host defense against pathogenic infection, but also the cornerstone of adaptive immune response. Upon pathogenic infection, pattern recognition receptors (PRRs) of host engage pathogen-associated molecular patterns (PAMPs) of pathogens, which initiates IFN production by activating interferon regulatory transcription factors (IRFs), nuclear factor-kappa B (NF-κB), and/or activating protein-1 (AP-1) signal transduction pathways in host cells. In order to replicate and survive, pathogens have evolved multiple strategies to evade host innate immune responses, including IFN-I signal transduction, autophagy, apoptosis, necrosis, inflammasome and/or metabolic pathways. Some avian viruses may not be highly pathogenic but they have evolved varied strategies to evade or suppress host immune response for survival, causing huge impacts on the poultry industry worldwide. In this review, we focus on the advances on innate immune evasion by several important avian immunosuppressive viruses (infectious bursal disease virus (IBDV), Marek’s disease virus (MDV), avian leukosis virus (ALV), etc.), especially their evasion of PRRs-mediated signal transduction pathways (IFN-I signal transduction pathway) and IFNAR-JAK-STAT signal pathways. A comprehensive understanding of the mechanism by which avian viruses evade or suppress host immune responses will be of help to the development of novel vaccines and therapeutic reagents for the prevention and control of infectious diseases in chickens.

## General Information About the Chicken Innate Immune Response to Viral Infection

Innate immunity is the first line of host defense against viral infection, playing an antiviral role through innate immune molecules (antimicrobial peptides and bacteriolytic enzymes), innate immune cells (phagocytes and non-phagocytic innate immune cells), and complement system (1, 2). At present, the mechanism, by which the engagement of PRRs with PAMPs activates the antiviral signal transduction pathway in hosts, attracts wide attention. One of the most important antiviral signal transduction pathways concerns type I interferon (IFN-I) signal transduction, regulating the expression of IFNs-I (IFN-α, IFN-β), members of the interferon family that are involved in antiviral immunity, anti-tumor immunity, and immunomodulation (3–6). Three types of PRRs are mainly responsible for switching on IFNs-I signal transduction pathway upon viral infection, including Toll-like receptors (TLRs), RIG-I-like receptors (RLRs) and cytosolic DNA sensors (7, 8). Once PRRs recognize PAMPs, the downstream adaptors will be recruited and activated, which leads to the activation and nuclear translocation of transcription factors, such as NF-κB, IRFs and AP-1, eventually inducing the production of IFN-I and exerting antiviral effects (9, 10). Secreted interferons induce the phosphorylation of Janus kinase 1 (JAK1) and tyrosine kinases 2 (TYK2) in adjacent cells by binding to interferon receptors on the surface of adjacent cells and then actives the signal transducers and activators of transcription 1 (STAT1) and STAT2 (11). The activated STATs translocate to the nucleus and further bind to IRF9, forming a trimer complex, which initiates the transcription of interferon-stimulated genes (ISGs) (11–14).

The engagement of viral PAMPs by chicken PRRs, which include TLRs, RLRs, and cytosolic DNA sensor cGAMP synthase-stimulator of interferon genes (cGAS-STING), activates IFN-I signal transduction pathway (7, 15). Ten types of TLRs exist in chicken (16), including ChTLR1A, chTLR1B, chTLR2A, chTLR2B, chTLR3, chTLR4, chTLR5, chTLR7, chTLR15 and chTLR21, which are primarily expressed in epithelial cells and immune cells (17). TLR3 and TLR7 mainly recognize viral RNA in endosomes, and the activated Toll interleukin-1 receptor (TIR) domain recruits and activates several adaptors including MyD88, TRIF, PI3K, etc, which lead to the phosphorylation of interleukin 1 receptor-associated kinase (IRAK) and the activation of TGF-β-activated kinase 1 (TAK1) and Tank-binding kinase 1 (TBK1) (18–20). Finally, the activated TAK1/TBK1 triggers the downstream NF-κB and IRFs signaling pathways to initiate the expression of IFN-I (18, 21). In addition, the activated TAK1 also activates JNK and P38, which initiate the AP-1 signal transduction pathway and induce the expression of IFN-γ (22). Although TLR8 and TLR9 are naturally absent in chicken, chTLR21, a homolog of TLR9 in chicken, can recognize viral single-stranded RNA (ssRNA) and double-stranded RNA (dsRNA) instead, further activating the downstream antiviral signal transduction pathway (23). Retinoic-acid-inducible gene 1 (RIG-I) and melanoma-differentiation-associated gene 5 (MDA5), two members of RIG-I-like helicase receptors (RLRs) family, sense viral dsRNA in cytosolic through helicase domain, leading to the production of IFNs in host cells (10, 24). Due to the genetic deficiency of RIG-I and IRF3 in chicken, chicken MDA5 is mainly responsible for sensing viral RNA and activating downstream mitochondrial antiviral signaling protein (MAVS)-NF-κB/IRF7 signal transduction pathway to initiate the expression of IFN-I (25, 26). Of note, chicken laboratory of genetics and physiology 2 (chLGP2), another member of RLRs, cannot activate MAVS independently without caspase activation and recruiting domains (CARD) but promote nucleation of MDA5 oligomerization on dsRNA through chLGP2 end-binding (27). chMDA5 and chLGP2 sense viral RNA effectively and initiate antiviral signal transduction pathways, which compensate for the genetic deficiency of RIG-I. cGAS-STING, an important intracellular DNA receptor, can recognize intracellular viral dsDNA, catalyze 2’3’-cGAMP synthesis to activate STING located in the endoplasmic reticulum (28, 29). TBK1 and inhibitor of NF-κB kinase (IKK), recruited by activated STING, phosphorylates IRF7 or NF-κB to initiate the production of IFNs (30–33). chSTING can also act as an adaptor of chMDA5 to initiate chMDA5-chSTING signal transduction pathway, activating IRF7 and NF-κB, and inducing the production of IFNs (30). In addition, it was found that DEAD (Asp-Glu-Ala-Asp) box polypeptide 41 (DDX41), an important DNA sensor in human and mouse, can also sense viral DNA in chicken and trigger chDDX41-chSTING-IFN-β axis (34).

In order to survive and replicate in host, viruses have evolved varied strategies to evade host innate immunity. On the one hand, viruses evade type I interferon-mediated innate immune response *via* expressing viral proteins to target host proteins. On the other hand, viruses may express viral miRNAs or regulate host miRNAs expression to target host proteins, inhibiting host anti-viral response. In general, the immune evasion strategies of viruses include interfering with PAMPs recognition by host PRRs, inhibiting the activation of signal transducers MAVS and TBK1, affecting the phosphorylation of IRF, NF-κB and AP-1, ultimately suppressing IFN-I expression. In comparison with mammals, chickens have a special immune system. This review is mainly focused on the mechanism by which avian immunosuppressive viruses evade host PRRs-mediated signal transduction pathways, including IRF7/NF-κB signal transduction pathways and interferon-α/β receptor (IFNAR)-JAK-STAT signal transduction pathway.

## Innate Immune Evasion by Infectious Bursal Disease Virus (IBDV)

### Brief Introduction to IBDV

IBDV is the causative agent responsible for infectious bursal disease (IBD) in chickens. IBD, originally called Gumboro disease, is an acute, highly contagious and immunosuppressive poultry disease reported by Cosgrave as early as 1962 (35, 36). IBDV infection mainly causes severe apoptosis of proliferating B lymphocytes in the bursa of Fabricius (BF), eventually leading to immunosuppression (37). The immunosuppression in IBDV-infected chicken increases the risk of secondary infection or immune failure of subsequent vaccinations against other pathogens. Thus, IBD remains a big threat to the poultry industry across the globe. IBDV is a non-enveloped dsRNA virus, belonging to the genus *Avibirnavirus* in the family of *Birnaviridae* (35, 38). The viral genome is composed of two segments (segment A and segment B) of RNAs (39). In segment A, two overlapping open reading frames (ORFs) encode the non-structural viral protein 5 (VP5) and polymeric proteins that are hydrolyzed and processed into viral proteins VP2, VP3, and VP4 (40–42). Segment B encodes VP1, an RNA-dependent RNA polymerase (RdRp) (43, 44). Two serotypes of IBDV are identified based on virus neutralization test, including serotype I and serotype II, of which only serotype I can cause clinical diseases in chickens (45).

### IBDV Evades Innate Immune Response *via* Targeting Host Protein

**Figure 1:**
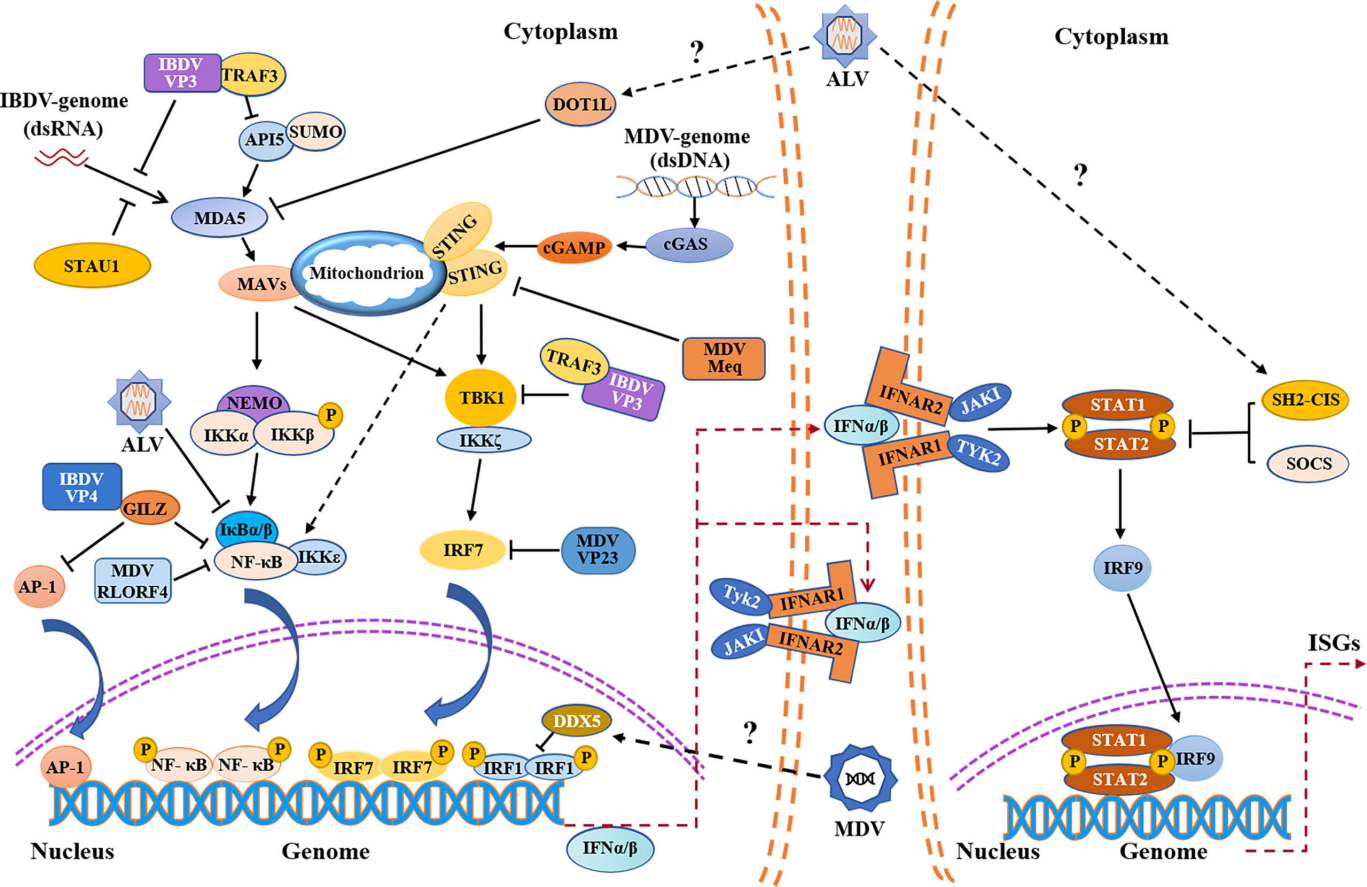
Schematic diagram of avian immunosuppressive viruses evading innate immune response in chicken. Evasion of the PRRs-mediated type I interferon signaling pathway and IFNAR-JAK-STAT signaling pathway by IBDV, MDV, ALV etc. IBDV, MDV and ALV exploit both viral and host proteins to evade innate immune response. IBDV and ALV mainly evade the innate immunity by inhibiting MDA5-mediated signaling pathway, while MDV evades the innate immunity by inhibiting cGAS-STING signaling pathway. IFNα/β promote the expression of ISGs by autocrine and paracrine action. MDA5, melanoma-differentiation-associated gene 5; API5, apoptosis inhibitor 5; TRAF3, tumor necrosis factor receptor associated factor 3; STAU1, staufen1; MAVS, mitochondrial antiviral signaling protein; NF-κB, nuclear factor-kappa B; NEMO, nuclear factor (NF)-kappaB essential modulator; IKKα, NF-kappaB kinase subunit alpha; IKKβ, nuclear factor kappa-B kinase beta; IκBα/β, nuclear factor kappa-B-α/β; IKKζ, nuclear factor kappa-B kinase zeta; IKKϵ, nuclear factor kappa-B kinase epsilon; AP-1, activating protein-1; GILZ, glucocorticoid-induced leucine zipper; DOT1L, disruptor of Telomeric silencing 1-like; cGAMP, cyclic GMP-AMP; cGAS, cyclic GMP-AMP synthase; STING, stimulator of interferon genes; TBK1, TANK-binding kinase 1; IRF, interferon regulatory transcription factor; IFNAR, interferon-α/β receptor; JAK1, Janus kinase 1; TYK2, tyrosine kinases 2; STAT, signal transducers and activators of transcription; SOCS, suppressor of cytokine signaling; SH2-CIS, Cytokine-inducible Src homology 2 -containing protein; ISGs, interferon-stimulated genes; P, phosphate; SUMO, SUMOylation.

Based on the experimental evidence available so far, RIG-I is naturally absent in chickens, thus viral dsRNA in the cytoplasm is mainly recognized through chMDA5 (25, 46). Upon IBDV infection, viral dsRNA is recognized by chMDA5, which triggers the downstream signal transduction pathway and induces the production of IFN-I (47). To avoid being recognized by chMDA5, IBDV has evolved with varied strategies to promote its survival and spread in host (**Figure 1**). It was reported that IBDV VP3 inhibited the expression of IFN-β by competitively binding to chMDA5 with virus dsRNA, achieving immune evasion (48). Similarly, IBDV VP3 also indirectly attenuates the chMDA5-mediated type I interferon signal transduction by regulating the modifications of host protein. It was found that apoptosis inhibitor 5 (API5) is a nuclear protein with anti-apoptosis function, which plays a role in regulating cell apoptosis (49). Recently, API5 was found to be a UBC9-dependent SUMOylated protein and was deSUMOylated upon IBDV infection, inhibiting chMDA5-dependent IFN-β induction. Specifically, IBDV VP3 inhibited the SUMOylation of API5 by targeting API5 and promoting UBC9-dependent proteasome degradation by binding to the ubiquitin E3 ligase TRAF3, ultimately leading to the reduction of chMDA5-dependent IFN-β production (49, 50). In addition to intervening the recognition of viral dsRNA genome by chMDA5, IBDV also employs other strategies to escape the innate immune response. It was reported that VP3 blocked the formation of TRAF3-TBK1 complex by reducing K33-linked poly-ubiquitination of Lysine-155 on TRAF3, inhibiting the production of IFN-I and facilitating viral replication (51). Interestingly, our previous study indicated that VP3 could interact with chicken Ribosomal Protein L18 (chRPL18) and chicken double-stranded RNA-activated protein kinase (chPKR), which enhanced IFN-I expression and inhibited viral replication (52). It seems that the interaction between IBDV and the host could be very complex during IBDV infection, and other viral components may also play dual roles similar to that of VP3 (53).

IBDV proteins can also target transcription factors NF-κB and AP-1 to suppress the expression of type I interferon (54) (**Figure 1**). Our previous study showed that IBDV VP4 could interact with glucocorticoid-induced leucine zipper (GILZ), a member of the glucocorticoid-responsive molecule with a proline-rich region (PER) at C-terminus that binds to the P65 subunit to inhibit the activity of NF-κB, thereby inhibiting the innate immune response in host (55). Furthermore, our data show that IBDV VP4 interacted with GILZ to inhibit its K48-Linked ubiquitylation, thereby inhibiting GILZ degradation. As a consequence, the accumulation of GILZ suppressed the NF-κB signal transduction pathway, leading to the inhibition of IFN-I production and promoting viral replication (54, 56). In addition, the accumulation of GILZ may also prevent AP-1 from activating the transcription of antiviral genes, leading to innate immunosuppression (57).

Regulation of host protein expression by IBDV to escape the innate immune response is also an important strategy for viral survival. IBDV can interfere with PRR recognition of viral dsRNA by targeting host proteins. Staufen1, a member of dsRNA binding proteins in host, was found to bind to viral RNA of certain viruses to facilitate viral replication such as human immunodeficiency virus (HIV-1), Influenza A virus (IAV), and hepatitis C virus (HCV) (58–61). Recently, it was reported that Staufen1 could competitively bind to viral dsRNA with chMDA5 upon IBDV infection, which inhibits chMDA5-mediated IFN-β production (62) (**Figure 1**), suggesting that host protein Staufen1 and IBDV VP3 may play a synergistic role in preventing chMDA5 from recognizing viral dsRNA upon IBDV infection, suppressing host response and favoring IBDV replication.

In comparison with IBDV VP3 and VP4, there are no reports currently available regarding the direct regulation of type 1 interferon signaling pathway by other IBDV viral proteins such as VP1, VP2 and VP5. It was found that VP2 and VP5 played important roles in assisting virus release by inducing apoptosis (63–67). As IBDV is a non-enveloped virus, it induces apoptosis and/or autophagy in host cell to promote its release and spread (68). It was reported that VP2 could interact with heat shock protein 90 (HSP90AA1) to activate autophagy at the immediately early stage upon infection, affecting IBDV replication, and then this autophagic response was inhibited thereafter (63). It should be noted that VP3 links PIK3C3-PDPK1 complex to AKT-MTOR pathway and inhibits autophagy, regulating viral replication (69). Thus, IBDV has developed some strategies to modulate autophagy to facilitate its survival and replication. In addition to inducing host autophagy, IBDV VP2 degrades the oral cancer overexpressed protein 1 (ORAOV1), an anti-apoptotic protein in host cells, inducing apoptosis and promoting virus release (64). Interestingly, IBDV VP5 has been established as a protein of both pro-apoptotic and anti-apoptotic functions, playing a dual role in IBDV-induced apoptosis. In the early stage of IBDV infection, VP5 inhibits apoptosis by interacting with PI3K P85α to facilitate viral replication (66), while in the late stage of IBDV infection, VP5 interacts with VDAC2 and RACK1 to form the VDAC2-VP5-Rack1 complex, resulting in apoptosis with cytochrome C release, promoting the release of virus (65, 67). Thus, IBDV-induced apoptosis might be an important strategy for IBDV to escape the host innate immunity. IBDV VP1, an RNA-dependent RNA polymerase (RdRp) of IBDV (44), has not been reported as a viral component involved in innate immune evasion by IBDV.

### IBDV Evades Innate Immune Response by Regulating Host miRNA Expressions

**Figure 2:**
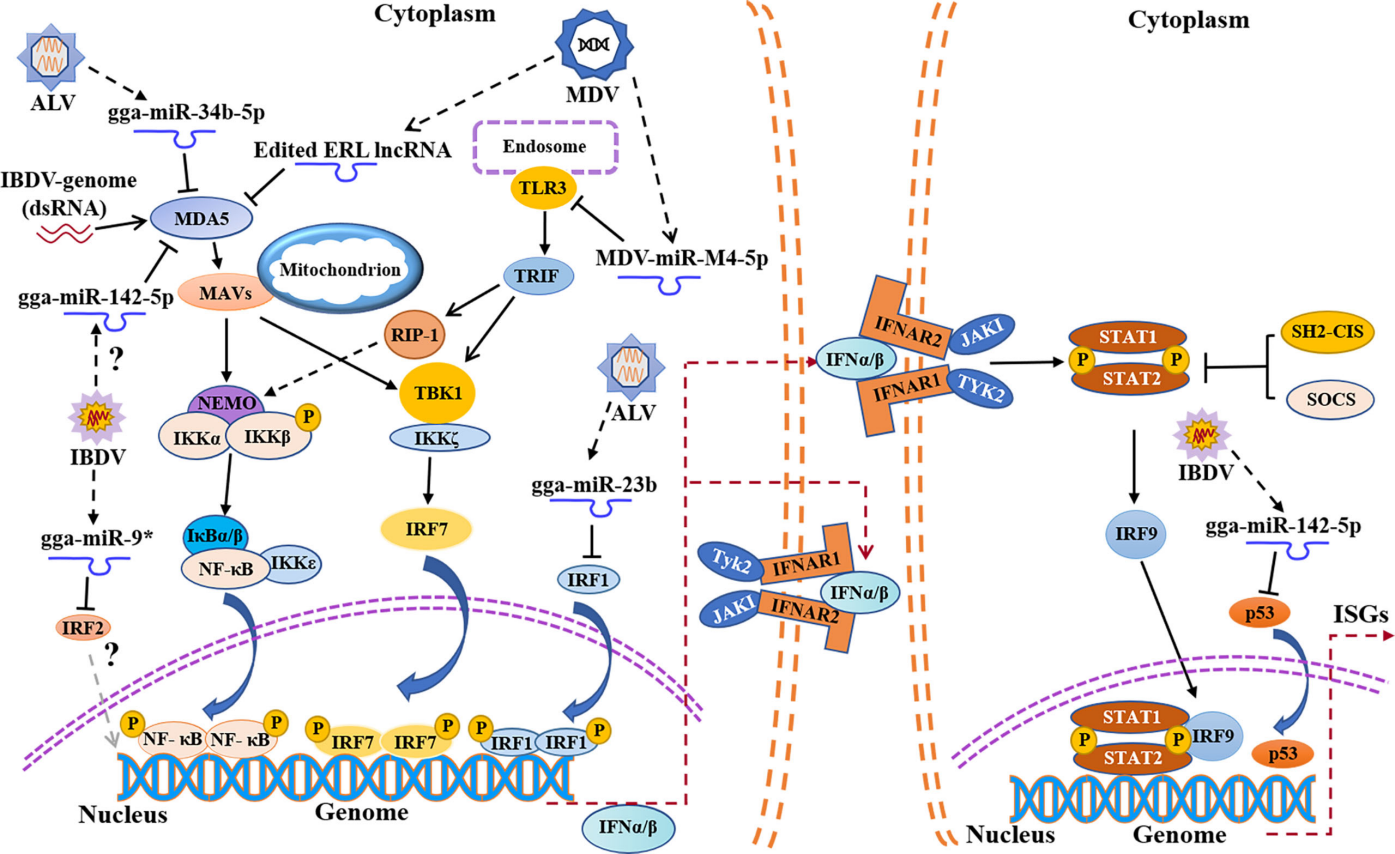
Schematic diagram of avian immunosuppressive viruses expressing viral miRNAs or regulating host miRNAs expression to suppress host innate immune response. TLR3, Toll-like receptors 3; TRIF, TIR domain-containing adapter-inducing interferon-β; RIP-1, receptor interacting protein-1.

MicroRNAs (miRNAs) are a family of small non-coding RNAs composed of 20-24 nucleotides, which play an important role in many biological processes by affecting the degradation and translation of target mRNAs (70, 71). Lines of evidence indicate that some miRNAs are involved in innate immune evasion of viruses (72, 73). Our previous data showed that 296 miRNAs were differentially expressed during IBDV infection (74). It was found that several miRNAs were closely related to immune evasion upon IBDV infection, such as gga-miR-9 (75), gga-miR-2127 (76), and gga-miR-142-5p (77) (**Figure 2**). Specifically, gga-miR-9 targeted IRF2 to inhibit the production of IFN upon IBDV infection, causing innate immune evasion (75). Since IRF2 was originally described as a transcriptional repressor (78), it may play a dual role in genes transcription (79, 80). P53, a tumor suppressor, plays an important role in innate immune regulation (81, 82). During IBDV infection, the expression and activity of chicken P53 (chp53) significantly increased, which upregulated the expression of a number of antiviral genes (IPS-1, IRF3, PKR, OAS and Mx), leading to the inhibition of IBDV replication (76). However, gga-miR-2127 down-regulates chp53 mRNA translation and inhibits innate immune response by targeting chp53 3’UTR (76), suggesting that IBDV might evade host response by manipulating the expression of chp53. In addition, it was reported that gga-miR-142-5p directly targeted the 3 ‘untranslated region of chMDA5 to attenuate IRF7-mediated innate signal transduction and facilitated IBDV replication (77). Thus, it seems that IBDV escapes the innate immune response not only at a protein level but also at an RNA level, which favors its survival and replication in host cells. No doubt, elucidation of the mechanisms will be of help to the development of novel vaccines and therapeutic reagents for the prevention and control of IBD.

## Innate Immune Evasion by Marek’s Disease Virus (MDV)

### Brief Introduction to MDV

Marek’s disease virus (MDV) is a highly oncogenic alpha-herpesvirus that causes lymphoid hyperplasia and lymphoma, eventually leading to death or immunosuppression in infected chickens (83, 84). MDV belongs to the genus *Mardivirus* in the family of *Herpesviridae*, which includes three serotypes, MDV serotype 1, 2 and 3. MDV serotype 1 (Gallid Herpesvirus 2, GAHV-2) was pathogenic to the chickens, while neither MDV serotype 2 (Gallid Herpesvirus 3, GAHV-3) nor MDV serotype 3 (Herpesvirus of Turkeys, HVT) was pathogenic to poultry (85). MDV genome is composed of a single linear dsDNA molecule with a length of about 180kb (86, 87). The structure of MDV virion is similar to that of other alpha-herpesviruses consisting of envelope, tegument, capsid and core. The viral core (viral DNA) is surrounded by an icosahedral symmetric capsid, and further surrounded by tegument and envelope (88).

### MDV Evades Innate Immune Response by Targeting Host Proteins

Current evidence suggests that the viral evasion of host innate immune response is the basis of viral survival and replication (89, 90). It was reported that the mRNA and protein expressions of IFN-α and IFN-β in the thymus and bursa of Fabricius of SPF chickens infected with virulent MDV decreased significantly in the lytic infection stage compared with that of the control group, indicating that MDV can inhibit IFN-I expression in host, resulting in immunosuppression (91). cGAS-STING, known as an important cytosolic DNA sensor for recognizing pathogenic DNA, undoubtedly plays an important role in host response to MDV infection (29). However, in order to survive and replicate better, MDV has evolved with varied strategies to antagonize cGAS-STING-mediated innate immune response in host (**Figure 1**). It was reported that five MDV proteins (Meq, RLORF4, US3, UL46 and VP23) had been identified to inhibit the production of IFN-I by regulating the cGAS-STING signal pathway (89). Meq, a major oncogenic protein of MDV, is involved in lytic infection, and is essential for the transformation of lymphocyte *in vivo* (92, 93). It was found that Meq bound to STING and IRF7, and then interrupted the formation of STING-TBK1-IRF7 complex, which inhibited the expression of IFN-β in chicken embryo fibroblast (CEF) with MDV infection (89). RLORF4, another viral protein encoded by MDV, is closely related to the pathogenicity of MDV (94, 95). It was found that RLORF4 could significantly inhibit cGAS-STING-mediated IFN-β production in host. Specifically, RLORF4 inhibited the translocation of NF-κB from cytoplasm to nucleus by binding to the Rel homology domains (RHD) of P65 and P50 subunits, ultimately inhibiting the expression of IFN-β. In addition, RLORF4 was also found to suppress tumor necrosis factor α (TNF-α)-induced activation of NF-κB, further inhibiting the production of IFN-I and promoting viral replication (96). Furthermore, it was reported that VP23 could interact with IRF7 to block the binding of TBK1 to IRF7, inhibiting the phosphorylation of IRF7, resulting in immunosuppression (90). Up to now, the specific role of US3 and UL46 expressed by MDV in escaping the innate immune response has not been clarified, but a recent publication provided a detailed review on the mechanism by which US3 and UL46 expressed by HSV-1, another member of the *Alphaherpesviridae*, inhibited the production of IFN-I and achieved innate immune evasion (97), which may provide some reference for exploring the mechanism of innate immune evasion of MDV. Of note, it is not clear whether MDV inhibits the innate immune response in host by suppressing the expression or function of TLRs *via* viral components.

Similar to IBDV, MDV can also inhibit innate immune response by hijacking host proteins (**Figure 1**). Dead-box Helicase 5 (DDX5), a member of DEAD Box family of RNA helicase, is involved in many physiological processes such as RNA metabolism, cell proliferation, apoptosis, and has also been found to be highly expressed in malignant tumor tissues (98–100). It was shown that DDX5 could be hijacked by viruses to promote viral replication (101, 102), and a similar phenomenon was found in MDV-infected host cells (103). It was reported that DDX5 could be hijacked by MDV in CEFs, resulting in increased expression and aggregation of DDX5 in the nucleus, which led to the downregulation of IRF1 and inhibited the IRF-1-mediated innate immunity (103, 104). It seems that DDX5 can be employed by virus for its survival and replication, providing a new insight into the mechanism by which viruses hijack host proteins to achieve innate immune evasion.

### Involvement of miRNAs in Innate Immune Evasion by MDV

MDV, a DNA virus, not only expresses its own microRNA against the innate immune response, but also utilizes host miRNAs to achieve immune evasion (**Figure 2**). It was reported that the expression of TLR3 in CEFs was upregulated post MDV infection, which further activated the innate immunity (105), but MDV appeared to employ special strategies to escape the innate immunity (106). MDV1-miR-m4-5p, a homolog of host miR-155, encoded by MDV genome, could target TLR3 and inhibit TLR3-mediated innate immune response, favoring viral replication (107, 108). It was reported that the chicken gga-miR-155-5p and MDV1-miR-m4-5p activate the JAK/STAT signal pathway by targeting suppressor of cytokine signaling 1 (SOCS1), increasing the expression of Adenosine deaminase acting on RNA1 (ADAR1), which hyperedited the edited repeat-long, long noncoding RNA (ERL lncRNA), a natural antisense transcript (NAT) of MDV protein meq (109, 110). The hyperedited ERL lncRNA further binds to MDA5, inhibiting the activity of MDA5 and resulting in immunosuppression (109). It was reported that MDV1-miR-M3 suppressed cisplatin-induced apoptosis by targeting SMAD2, a critical component in the transforming growth factor (TGF) β signal pathway, thus creating a cellular environment favorable to virus replication (111). Similarly, virus-encoded miR-M2-5p promotes cell viability and inhibits cell apoptosis through RBM24 and MYOD1-mediated signaling pathways (112). It seems that MDV modulates host response by self-encoded miRNAs targeting cellular proteins to favor viral survival and replication during viral infection.

## Innate Immune Evasion by Avian Leukosis Virus (ALV)

### Brief Introduction to ALV

ALV, an enveloped RNA virus belonging to the genus *Alpharetrovirus* in the family of *Retroviridae*, causes neoplastic diseases in chicken such as lymphoblastic leukemia, myeloblastic leukemia and erythroblastic leukemia, leading to the decline of egg production or even death (113). According to the characteristics of viral envelope proteins, ALVs isolated from chickens were divided into seven subgroups, A, B, C, D, E, J and K. Among them, ALV-E belongs to endogenous retroviruses and the others are exogenous retroviruses (114, 115). ALV genome is composed of two copies of ssRNA, harboring three genes encoding important structural proteins, gag, pol and env. *Gag* gene encodes several non-glycosylated proteins, including viral capsid protein P27, matrix protein P19 and nucleocapsid protein P12, *pol* gene encodes reverse transcriptase and integrase p32, and *env* gene encodes surface protein GP85 and transmembrane protein GP37 (116, 117).

### ALV Evades Innate Immune Response by Targeting Host Proteins

It was reported that the viral RNA of ALV could be recognized by TLR7 and MDA5 in host cells with ALV infection, inducing the expression of ISGs and cytokines (118). However, the information regarding the mechanism by which ALV evades the host innate immune response is quite limited (**Figure 1**). Cytokine-inducible Src homology2 (SH2)-containing protein (CIS), a member of the SOCS family, can negatively regulate innate immune response and promote viral replication (119). Recently, it was found that the expression of CIS was up-regulated in DF-1 cells with ALV-J infection, inhibiting the expression of IFN-I and ISGs, while the expressions of IFN-I and ISGs significantly increased in CIS^-/-^ DF-1 cells with ALV-J infection (120). Thus, CIS may be an important factor that helps ALV-J evade the innate immune response in host. Similarly, it has recently been shown that ALV-J infection could inhibit the phosphorylation of the JAK2/STAT3 signaling pathway by upregulating the expression of SOCS3, which represses host innate immunity and facilitates viral replication (121). In addition to directly targeting negative regulators for interferon signal transduction pathways to achieve innate immune evasion, ALV-J can also suppress the expression of IFN by targeting host protein indirectly (122, 123). Disruptor of Telomeric silencing 1-like (DOT1L), a histone methyltransferase, has been proven to catalyze the methylation of histone H3 lysine79 and plays a role in the development of malignant tumors including pancreatic cancer, leukemia and breast cancer (124–126). Recently, it was proposed that DOT1L was also involved in innate immune response and acted as an antiviral regulator during ALV-J infection (127, 128). Interestingly, it was found that the expression of DOT1L was up-regulated in HD-11 cells with ALV-J infection, inhibiting the expression of MDA5 and impairing the activation of type I interferon signal transduction pathway, which indicates that DOT1L plays a positive role in viral replication (129). It seems that the antiviral effect of DOT1L may vary according to the types of viruses. Meanwhile, in view of the facts that DOT1L knockout does not affect IFN-I expression after poly(I:C) stimulation (129), DOT1L may be involved in the innate immune evasion by ALV *via* affecting the recognition of ALV RNA by host PRRs, but direct experimental evidence needs to be obtained. In addition, it was found that ALV-J attenuated IFN-I production by blocking phosphorylation of IκB and inhibiting the expression of NF-κB (123), but it was unclear which viral protein affected IκB phosphorylation. Furthermore, p53 is also involved in host response to ALV infection. It was found that p53 could prevent ALV-J from resisting antiviral innate immunity, indicating the importance of p53 in host response against ALV-J (130). Although some studies suggest that p53 inhibits viral replication by affecting innate immune factors, the potential association between chp53 and those innate immune factors remains unclear (76, 130). However, a recent study indicates that p53 recruits the histone deacetylase 1 and 2 (HDAC1/2) complex to the ALV-J long terminal repeat (LTR) region which regulates viral replication to switch off gene expression (131, 132), suggesting that p53 inhibits ALV replication not only by activating innate immune response but also by inhibiting ALV-J LTR activity. Current information is still insufficient to determine the proportion of chp53 effect on suppression of ALV-J replication *via* innate immunity or regulating LTR of ALV. Up to now, it is still unclear whether ALV suppresses host response by affecting p53-mediated signaling pathways *via* interaction with host cells at a protein level. More studies will be required to determine the crucial viral proteins of ALV that target cellular proteins involved in signaling pathways of host innate immune response.

### Involvement of miRNAs in Innate Immune Evasion by ALV

In addition to interference with the expression of MDA5 by DOT1L, ALV also impairs the activation of MDA5-mediated IFN-I signal transduction pathway by regulating the expression of host microRNAs (**Figure 2**). It was reported that the expression of miR-34b-5p significantly increased in the spleen and thymus of ALV-J-infected chickens, which inhibited the expression and activation of MDA5, facilitating ALV-J replication (133). Similarly, the expression of miR-23b also increased upon ALV infection, and miR-23b directly targeted IRF1, thereby reducing the expression of IFN-β (134). In comparison with IBDV or MDV, few reports are currently available regarding innate immune evasion by ALV. Considering the unique genomic structure of ALV as a retrovirus and its persistent infection in host cells, it is highly possible that ALV achieves innate immune evasion by more than just blocking signal transduction pathway for type I IFN expression. More efforts will be required to elucidate the mechanism of innate immune evasion by ALV.

## Innate Immune Evasion by Other Avian Immunosuppressive Viruses

Avian reovirus (ARV) belongs to the genus of *Reoviridae* in the family of *Reoviridae*, and the viral genome is composed of dsRNA (135–137). Previous studies demonstrated that ARV could cause damages to lymphatic organs such as bursa of Fabricius, thymus and spleen, resulting in immunosuppression and reduced immune response to other secondary infections (138, 139), but the research on the mechanism by which ARV evades innate immune response is lacking. It was found that σA could bind to dsRNA irreversibly, which inhibited the activation of dsRNA-dependent protein kinases, reducing the production of IFNs to resist the antiviral response in host (140, 141). Recently, it was reported that MDA5 and TLR3 were involved in the recognition of ARV (142–144), but it remains unclear whether ARV evades innate immune response by affecting this recognition. In addition, the role of miRNAs in innate immune evasion by ARV remains unclear.

Reticuloendotheliosis virus (REV), a member of the genus *γ-retrovirus* in the family of *Retroviridae*, can cause the atrophy of thymus and bursa of Fabricius in chickens, resulting in immunosuppression (145). Some viruses have evolved strategies to hijack the exosomes for immune evasion (146, 147). It was recently reported that the exosomes were more efficiently employed by REV to inhibit the expression of innate immune factors compared with the infection by free REV virions (148).It was reported that expressions of TLR4 and TLR7 significantly decreased during REV infection (149), suggesting that REV may evade innate immune response by inhibiting the expression of PRRs in host, but the specific mechanism remains to be investigated.

As for chicken infectious anemia virus (CIAV), another important avian immunosuppressive virus widely distributed in flocks across the globe, there are only few reports regarding the decreased number and activity of NK cells and macrophages in chickens (150, 151). More studies are encouraged to investigate the mechanism by which CIAV evades innate immune response.

## The Differences Between Avian Influenza Virus (AIV) and IBDV in Innate Immune Evasion

Innate immune evasion is not only the tactics employed by immunosuppressive virus for persistent infection but also the strategy used by non-immunosuppressive virus to survive early during infection. It would be intriguing to compare the differences between IBDV, a typical avian immunosuppressive virus, and AIV, an important avian non-immunosuppression virus, in innate immune evasion. AIV belongs to the genus of influenza A virus in the family of orthomyxoviridae (53). The viral genome of AIV is composed of negative-stranded RNA, which encodes glycoproteins HA and NA, matrix proteins M1 and transmembrane proteins M2, polymerase proteins PB1, PB2 and PA, and nucleoprotein NP (152).

Similar to IBDV, AIV has also developed the immune escape strategy by inhibiting the activation of the chMDA5-chMAVS pathway (27, 153, 154). It was reported that AIV NS1 and PB1 acted in concert to antagonize chicken type I IFN secretion in HD-11 cells (153, 155–157), and NS1 could interact with TRIM25, a member of E3 ubiquitin ligase, to inhibit IFN-β expression by regulating ubiquitin-proteasome degradation pathway (158). It seems that the ubiquitin-proteasome degradation pathway could be utilized by both avian immunosuppressive viruses and non-immunosuppressive viruses to escape the innate immunity. In addition, it was found that IBDV could induce autophagy and destroy autophagosomes to promote viral maturation and release (159), which suggests that IBDV-induced autophagy might be related to the innate immune evasion by IBDV. In comparison, AIV PB1 can induce mitophagy, thus affecting the innate immune response mediated by chMAVS to achieve innate immune evasion to favor its replication (160–162). Taken together, IBDV infection is characterized by the damages to the bursa of Fabricius where B lymphocytes develop and mature, leading to significant immunosuppression, which greatly increases the risk of secondary infection with other pathogens, whereas AIV infection evades innate immunity early during infection and subsequently causes a strong innate immune response in host, eliciting severe inflammatory responses (163–165). Further investigation into the mechanism of innate immune evasion by avian immunosuppressive virus and non-immunosuppressive virus will provide valuable reference to the understandings of pathogenesis of viral infection.

## Conclusion

Viruses can evade detection or clearance by both innate and adaptive immune responses, while their strategies of evading innate immune responses are extraordinarily complex. Although there is a considerable advance in the understandings of the innate immune evasion by avian viruses that was mainly attributable to the results from those studies on host PRRs, the IRF7/NF-κB signal transduction pathway and the IFNAR-JAK-STAT signal transduction pathway during viral infection, little information is available regarding the innate immune evasion by avian viruses *via* pyroptosis, necroptosis, epigenetic regulation and other apparent metabolic pathways. Thus, studies on these aspects will be highly encouraged.

Infections of flocks by avian immunosuppressive viruses not only cause death in chickens, but also increase the susceptibility of the survivals to subsequent pathogenic infections due to the compromised immune response in infected chickens. The immunosuppressive viruses might directly attack immune organs and cells. For instance, IBDV induces chicken immune organ atrophy and apoptosis by targeting proliferating B cells in the bursa of Fabricius, MDV infection leads to early cytolytic infection of B cells and transformation of T cells and then induces proliferative lesion in chicken immune organs, and ALV induces several types of cellular tumors, such as lymphocytoma and intravascular lymphoid leucosis involving lymphoidocytes. These viruses cause damages to the immune system, leading to immunosuppression in the survivals of the infected chickens. Obviously, these avian immunosuppressive viruses (IBDV, MDV, ALV, etc.) suppress host immune response by either interaction of viral proteins with host cellular proteins or by regulating microRNAs expression. Thus, the immune suppressive state of virus-infected chickens is the consequence of virus-host interaction at both protein and RNA levels. Further investigation into the strategies by which avian immunosuppressive viruses evade the innate immune response will help to better understand the pathogenesis of avian immunosuppressive virus infection and lay a foundation in future studies, eventually contributing to the development of effective measures for the prevention and control of avian immunosuppressive virus.

## Author Contributions

Conceived and designed: SZ; wrote the paper: HW and WL; revised the paper: SZ. All authors contributed to the article and approved the submitted version.

## Funding

This work was supported by grants from the National Natural Science Foundation of China (# 32130105) and Earmarked Fund for Modern Agro-Industry Technology Research System (#CARS-40), China.

## Conflict of Interest

The authors declare that the research was conducted in the absence of any commercial or financial relationships that could be construed as a potential conflict of interest.

## Publisher’s Note

All claims expressed in this article are solely those of the authors and do not necessarily represent those of their affiliated organizations, or those of the publisher, the editors and the reviewers. Any product that may be evaluated in this article, or claim that may be made by its manufacturer, is not guaranteed or endorsed by the publisher.
